# Hepatitis C Treatment Initiation During Hospitalization for People Who Use Drugs: A Narrative Review of the Literature

**DOI:** 10.1093/ofid/ofaf237

**Published:** 2025-04-24

**Authors:** Jacob Denkins, Jane Babiarz, YoungYoon Ham, HaYoung Ryu, Brian Ricci, Carissa Williams, Ian Alexander, Kendall Tucker, L Madeline McCrary, Ximena Levander

**Affiliations:** Department of Pharmacy, Oregon Health & Science University, Portland, Oregon, USA; Division of Internal Medicine and Geriatrics, Department of Medicine, Oregon Health & Science University, Portland, Oregon, USA; Department of Pharmacy, Oregon Health & Science University, Portland, Oregon, USA; Department of Pharmacy, Oregon Health & Science University, Portland, Oregon, USA; Division of Internal Medicine and Geriatrics, Department of Medicine, Oregon Health & Science University, Portland, Oregon, USA; Division of General Internal Medicine and Geriatrics, Department of Care Management, Oregon Health & Science University, Portland, Oregon, USA; Division of General Internal Medicine and Geriatrics, Department of Care Management, Oregon Health & Science University, Portland, Oregon, USA; Department of Pharmacy Practice, College of Pharmacy, Oregon State University, Portland, Oregon, USA; Division of Infectious Diseases, Department of Medicine, Washington University School of Medicine, St Louis, Missouri, USA; Section of Addiction Medicine, Division of General Internal Medicine and Geriatrics, Department of Medicine, Oregon Health & Science University, Portland, Oregon, USA

**Keywords:** hepatitis C virus, hospitalization, people who use drugs, transitional care, vulnerable populations

## Abstract

People who use drugs (PWUD) are increasingly acquiring and transmitting hepatitis C virus (HCV) and being admitted to the hospital for often costly non–HCV-related conditions. Traditionally, treatment of HCV has been deferred to the outpatient setting. However, outpatient HCV follow-up can be an arduous process to navigate with resultant gaps in care, especially for PWUD with numerous complex psychosocial and medical comorbidities. Hospitalization presents a key opportunity to initiate treatment, and several institutions have piloted inpatient treatment models with intensive outreach. We conducted a narrative review of peer-reviewed literature (2014–2024) evaluating hospital-based HCV treatment models for PWUD; 6 studies from 4 countries met inclusion criteria. Evidence suggests that engaging PWUD during hospitalization leads to higher treatment initiation and completion as compared with standard-of-care outpatient referral. Inpatient HCV treatment models should be one part of a comprehensive plan in the United States and internationally to eliminate HCV for all.

The Viral Hepatitis National Strategic Plan and the World Health Organization 2030 Global Elimination goals outline 3 core indicators of progress toward hepatitis C virus (HCV) elimination [1, 2]: (1) reducing the rate of new HCV infections by 90%, specifically among people who use drugs (PWUD); (2) increasing the proportion of people with HCV clearance by 80%; and (3) reducing the rate of HCV-related deaths by 65% [2]. Injection drug use accounts for nearly half (43.6%) of total global HCV incidence, and progress toward HCV elimination remains far below the target of 80% [2]. An analysis demonstrated that the cost of expanding HCV treatment among the publicly insured in the United States would result in HCV-related cost savings of nearly $200 000 cumulatively per patient and national net savings of >$49 billion over 10 years [3]. Treating and curing HCV among PWUD who are infected also reduces transmission to new people, a strategy for microelimination known as *treatment as prevention* [4, 5]. Considering the high proportion of PWUD in the global HCV burden, the health care savings associated with HCV treatment, and studies showing that treatment among PWUD acts as prevention, it is imperative to focus resources on improving diagnostic strategies and innovative care delivery models to achieve elimination.

The traditional model of HCV treatment is a multistep process: HCV antibody testing followed by confirmatory RNA testing, diagnosis disclosure, referral to provider (historically requiring specialist involvement), treatment consultation, medication initiation, treatment completion, and laboratory confirmation of cure [6]. This model harbors multiple barriers for PWUD, who are less likely to be linked to treating clinicians [7]. Systemic barriers to accessing treatment include insurance or financial challenges, insufficient health care providers in rural areas, long wait times to establish care, and stigma and discrimination surrounding substance use, including misconceptions among providers about treatment eligibility and accessibility for PWUD. Some clinicians may mistakenly believe that PWUD should not be treated due to patient complexity, leading them to discourage or withhold treatment, further limiting access [8]. Barriers specific to HCV treatment include delayed diagnosis in the 2-step antibody-RNA testing algorithm and insurance prior authorizations, as well as lack of colocalization of HCV treatment with services such as addiction, harm reduction, and mental health care. In addition, stigma may contribute to delaying or avoiding care and loss to follow-up [9]. Complex medical and psychosocial comorbidities and lack of housing, telecommunication, and transportation resources also make traditional referrals to providers challenging [10].

Equipping high-impact care settings, such as health care centers for the unhoused, syringe service programs, street medicine initiatives, substance use disorder (SUD) treatment programs, and criminal-legal/correctional settings, with the means to engage PWUD in HCV treatment are demonstrated strategies for increased engagement and successful treatment [11–14]. Telehealth-based HCV treatment in partnership with SUD programs provides high treatment initiation and completion as compared with traditional models [15–17]. HCV-based interventions for PWUD during hospitalization, or inpatient admission, have been put forward as potential opportunities to engage patients who are not connected to outpatient health care, particularly given increasing rates of hospital admissions among people with SUD [18, 19]. However, HCV treatment initiation during hospitalization can face challenges: first, around medication procurement with insurance coverage limitations, especially for US patients from non-Medicaid expansion states; second, from patients who may have conflicting psychosocial needs or other prioritizations when recovering from acute illness [20, 21]. Given the need to develop novel evidence-informed interventions to address ongoing HCV elimination gaps, we review models that inform best practices and future directions for using the inpatient setting as an opportunity to engage PWUD in HCV treatment.

## METHODS

### Search Strategy

**Figure 1.**
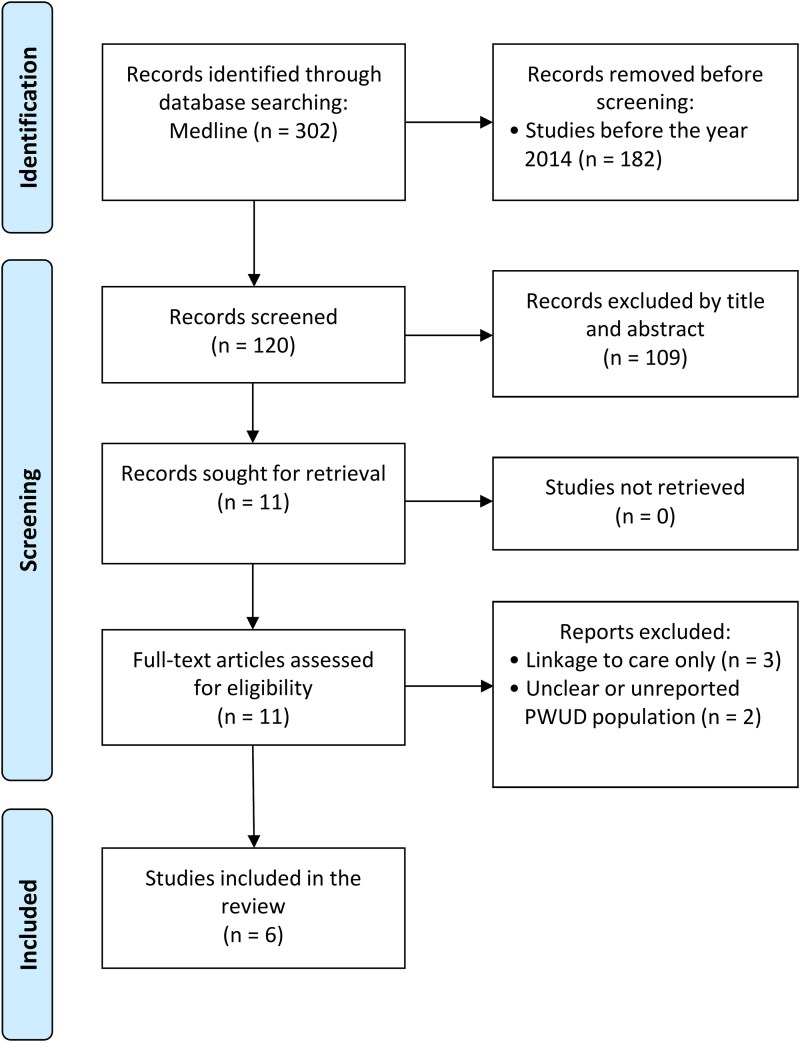
PRISMA flow diagram. PWUD, people who use drugs.

We searched Ovid Medline using a combination of MeSH terms for articles published between 2014 and 2024 in collaboration with Andrew Hamilton, MS/MLS, a research librarian at Oregon Health & Science University, who assisted in developing and refining the search strategy. The article selection process is illustrated in Figure 1 in a PRISMA flow diagram. Keyword searches combined common terms for HCV, SUD, and inpatient admission. The search included variations of “hepatitis C,” “hepacivirus,” “substance use disorder,” “intravenous drug use,” “IV drug use,” “hospitalization,” “inpatient,” “admission,” and “discharge.” Boolean and adjacency operators were used to refine the results and allow for contextual variation in phrasing. Our final search strategy consisted of 26 queries, systematically combining subject headings and keyword searches. The search identified 302 articles, which were further limited to studies published between 2014 and 2024, yielding 120 results for abstract screening.

### Inclusion Criteria

Articles were included if inpatient identification and treatment of HCV were the focus of the intervention for adult patients with history of or active SUD. Articles had to be written in English; we included studies from any country. Articles were excluded if they focused on other infectious processes only (eg, HIV), if the intervention was limited to an outpatient referral (considered standard-of-care/traditional HCV treatment model), if the focus was exclusively on improving inpatient HCV screening, if the intervention did not focus on or specifically address PWUD, or if the study was observational (nonintervention).

### Data Extraction

An overall 120 articles were identified, and abstracts were reviewed and screened for inclusion by at least 2 authors. After initial abstract review, 11 articles underwent full-text review for study inclusion. The authors adjudicated as a group when necessary for borderline cases. Six articles were included for full abstraction in the narrative review. Relevant data were extracted with a standardized Excel spreadsheet. When data of interest were not available in the article, we reached out to study authors in attempts to clarify or provide further details.

## REVIEW OF INPATIENT HCV TREATMENT MODELS

The 6 studies reviewed provide insights into the efficacy of initiating direct-acting antiviral (DAA) treatment during hospitalization for PWUD. These studies explore different models of hospitalized patient treatment initiation, assessing treatment uptake, completion rates, sustained virologic response, and barriers to care. Table 1 summarizes the findings of the articles selected for review.

**Table 1.** Treatment Models

First Author and Year	Description: Departments Involved	Study Design, Sample,^c^ and Setting	DAA Initiated: All Enrolled,^a^ Inpatient,^b^ Outpatient	Treatment Completed: All Enrolled,^a^ Inpatient Initiators, Outpatient Initiators	SVR12 Confirmed: All Enrolled,^a^ All DAA Initiators	DAA Access	Key Features
Midgard 2024 [22]	Immediate inpatient HCV assessment and DAA initiation vs outpatient referral RCT, primary outcome was treatment completion at 6 mo, outpatient support at a low-threshold HCV clinic • Addiction medicine, internal medicine, psychiatry	Stepped-wedge RCT; N = 200, n = 102 inpatient arm, n = 98 outpatient arm; PWUD: 92%; Oslo, Norway; multicenter	80 (159/200) 87 72	71 (141/200) 68 (67/98)^d^ 35 (36/102)^d^	63 (126/200)^e^ 79 (126/159)^e^	Unrestricted access to DAAs in Norway	RCT comparing early assessment and initiation vs standard of care; collaboration with local low-barrier HCV clinic
McCrary 2023 [23]	Addiction medicine team identified patients, ID specialists initiated treatment inpatient or postdischarge with telehealth • Addiction medicine, ID	Quality improvement program evaluation, N = 28, PWUD: 100%, North Carolina, academic medical center	68 (19/28) 11 8	46 (13/28) 73 (8/11) 63 (5/8)	43 (12/28) 63 (12/19)	Outpatient specialty pharmacy delivered for inpatient initiators; manufacturer's assistance program	Multidisciplinary; e-consult early assessments; telehealth; structured admission-to-SVR workflow
Babiarz 2024 [24]	Multidisciplinary HCV treatment program, electronic surveillance patient identification, telehealth consultation, inpatient or postdischarge DAA initiation • Addiction medicine, case management, ID, internal medicine, pharmacy	Prospective cohort, N = 25, PWUD: 100%, Oregon, academic medical center	76 (19/25) 8 11	56 (14/25) NR NR	44 (11/25) 59 (11/19)	Hospital's outpatient pharmacy; prior authorizations not required for DAAs in Oregon	Telehealth consultation with outpatient DAA-prescribing provider; multidisciplinary; frequent telehealth follow-up; prepaid phones and respite housing; financial incentives
Perera 2024 [25]	Implemented electronic medical record best practice alert, immediate DAA initiation or linkage to care postdischarge • Ottawa Hospital Viral Hepatitis Program	Quality improvement program evaluation, N = 22, PWUD: 82%; Ottawa, Canada; academic medical center	73 (16/22) 11 5	59 (13/22) NR NR	45 (10/22) 77 (10/16)	Unrestricted access to DAAs in Ontario, Canada	Best practice alert system; preestablished hepatitis program
Chiong 2019 [26]	HCV screening during hospitalization, DAAs initiated at discharge, outpatient follow-up via generalists or telephone outreach • ID	Prospective feasibility study, N = 70, PWUD: 80%; Sydney, Australia; tertiary referral center	66 (46/70) 21 25	56 (39/70) 95 (20/21) 76 (19/25)	53 (37/70) 80 (37/46)	Subsidized after HCV genotyping in Australia	HCV genotyping required for DAA initiation; summary letter to patients' generalist; outpatient initiation with generalists, ID specialists, or hepatologist
Le 2022 [27]	Multidisciplinary HCV screening on psychology and medicine wards, inpatient DAA initiation, follow-up at VA liver clinic • Case management, ID, internal medicine, pharmacy, psychiatry	Quality improvement program evaluation, N = 23, PWUD: 65%, California, VA medical center	100 (23/23) 23 0	91 (21/23) 91 (21/23) None	91 (21/23) 91 (21/23)	Hospital inpatient pharmacy; free or low cost for US veterans	Multidisciplinary; comprehensive VA system integration; excluded patients with significant concern for noncompliance; collaboration with local HCV clinic

Data are presented as % (No.) where shown.

Abbreviations: DAA, direct-acting antiviral; HCV, hepatitis C virus; ID, infectious disease; NR, not reported; PWUD, people who use drugs; RCT, randomized controlled trial; SVR, sustained virologic response; SVR12, SVR at 12 weeks posttreatment; VA, Veterans Affairs.

^a^Total number of patients with confirmed HCV infection (+ HCV RNA) who enrolled in the program to pursue treatment.

^b^Evaluated from the total number of participants included in the study. Some studies reported percentage of treatment completion and SVR over number of all DAA initiators.

^c^DAA initiated during hospitalization or at time of discharge.

^d^Within 6 months after study enrollment.

^e^SVR at 4 weeks posttreatment.

### Treatment Initiation

Several studies demonstrated higher treatment uptake when HCV treatment was initiated during hospitalization, while others focused on treatment engagement and completion without directly comparing inpatient vs outpatient initiation. Midgard et al examined treatment initiation in a stepped-wedge trial where hospital departments transitioned from referral-based outpatient care to immediate treatment initiation during hospitalization or as early as possible after discharge. Within 6 months, 86% of patients in the intervention group initiated treatment, as opposed to 46% in the standard-of-care group [22]. Similarly, McCrary et al, Babiarz et al, and Perera et al reported inpatient and outpatient treatment initiations within their study populations, showing an inpatient treatment initiation range of 39% to 76%, with notably lower outpatient initiation between 24% and 53% [23–25]. In contrast, Chiong and Post designed a model that did not initiate treatment during inpatient stay but instead at hospital discharge or in an outpatient setting once the patient was linked to care. The study compared these 2 groups, finding that 30% of patients were discharged with DAAs in hand, while 36% initiated treatment in an outpatient setting [26]. Le et al included only inpatient treatment initiators and did not have outpatient initiators for comparison [27].

### Treatment Completion and Sustained Virologic Response

Among studies that reported treatment completion for inpatient initiation (including those that started treatment right at the time of hospital discharge) and outpatient referral initiation, completion for the inpatient group ranged from 68% to 95%, whereas that for the outpatient group ranged from 35% to 76% [22, 23, 26]. Similarly, studies that reported sustained virologic response for both groups found that inpatient or discharge-based sustained virologic response at 12 weeks posttreatment (SVR12) ranged from 61% to 95%, while outpatient SVR12 ranged from 63% to 68% (not shown in Table 1) [22, 23, 26]. While Chiong and Post reported a slightly higher percentage of patients initiating treatment in outpatient settings, only 68% of those achieved SVR12 as opposed to 95% of those initiated at discharge [26].

### Shared Intervention

Across the 6 models, various interventions were implemented to improve treatment initiation, retention, and completion. Each model focused on early engagement and hospital-based treatment initiation, where treatment was started during hospitalization or at discharge, rather than relying on outpatient follow-up. Several studies also employed electronic and system-based identification of eligible patients to enhance treatment uptake, such as Perera et al, who integrated an electronic medical record alert system, and Barbiarz et al, who utilized an electronic clinical surveillance program to flag patients who were HCV positive for referral [24, 25].

Care coordination and discharge planning were key components of several treatment models. Each model mentioned some method of structured hospital-to-outpatient transitions, connecting patients to postdischarge follow-up care. Babiarz et al utilized a hospital-based telehealth treatment model to facilitate continuity of care postdischarge [24]. Several studies emphasized integration with other medical and social services to improve treatment retention. McCrary et al followed patients by telehealth, while Le et al engaged patients in psychiatric and residential treatment programs [23, 27].

## KEY FACTORS FOR A SUCCESSFUL HCV TREATMENT MODEL

### Compressing Time From Screening to Treatment

HCV screening and clinical assessments during hospitalization expedited patient treatment initiation. Le et al benefited from reflex HCV RNA testing, enabling immediate confirmation and prompt treatment starts [27]. Perera et al utilized a best practice alert system to facilitate early HCV antibody screening upon admission [25]. McCrary et al and Babiarz et al prioritized completing clinical evaluations during the hospitalization to reduce the losses to follow-up commonly seen with outpatient cases [23, 24]. Midgard et al reported a median time to treatment of 4.5 days in the intervention arm as opposed to 71 days in the outpatient control arm among those who initiated treatment within 6 months [22]. By contrast, Chiong and Post required genotyping after antibody testing, resulting in a median treatment initiation time of 59.5 days. The authors acknowledged that this delay in therapy shifted treatment to the outpatient setting and contributed to lower DAA initiation and completion [26].

### Prioritizing Inpatient DAA Initiation

Treatment models that initiated HCV treatment during hospitalization consistently demonstrated superior outcomes as compared with outpatient referral to treatment. In the Midgard et al study, 86% of patients in the inpatient arm initiated treatment within 6 months as opposed to 46% in the outpatient arm [22]. McCrary et al initially prescribed DAAs in only the outpatient setting, but after many patients neglected to initiate treatment, the model pivoted to initiate DAAs during hospitalization, improving uptake [23]. Le et al reported the only treatment model in which all patients started DAAs during hospitalization, with 91% achieving treatment completion. The authors suggested that part of this success may stem from patients recognizing the simplicity and tolerability of DAAs when initiated in a supportive monitored environment, motivating them to continue treatment postdischarge [27].

### Streamlined Access to DAAs

Ease of access to DAAs for hospitalized patients differed among health care systems and significantly influenced the success of inpatient HCV treatment models. Le et al had access to the US Department of Veterans Affairs health system, where DAAs were available on inpatient formularies, allowing for prompt initiation of therapy during hospitalization [27]. International models, such as those reported by Midgard et al in Norway and Perera et al in Canada, benefited from national health care systems that provided DAAs at no cost to patients [22, 25].

In contrast, major US hospitals initially faced challenges related to insurance and medication procurement processes for patients who were hospitalized. For the treatment models described by Babiarz et al and McCrary et al, DAAs were strict nonformulary items in the hospital and were unavailable through the inpatient pharmacy. To address the barrier, each system sought expertise and coordination with the pharmacy to design a workflow for DAA procurement. DAAs were prescribed and sent to the hospital's outpatient/specialty pharmacy, and prior authorization was obtained if needed. DAAs were then dispensed to the patient or HCV treatment team member and taken into the hospital as the patient's own medication per institutional policies [23, 24]. Unlike the US Veterans Affairs system or countries with single-payer health care system, the landscape for insurance barriers across US hospitals is rather challenging due to differences in DAA coverage for state/region-specific government-subsidized insurance (eg, Medicaid, Medicare) and private, commercial insurance plans. Understanding insurance-specific requirements with institutional policies for use of nonformulary medications such as DAAs may require collaboration with pharmacy and case management experts. For uninsured individuals, medication assistance programs through manufacturers were pursued, typically early during the hospitalization to minimize delays in treatment initiation [23, 24]. These logistical hurdles impeded starting therapy and created missed opportunities if patients were discharged prior to receiving medications.

The availability of pangenotypic DAAs also varied among models. In the Chiong and Post study conducted in Australia, the treatment protocol mandated HCV genotyping before starting therapy, which contributed to delays, as compared with models utilizing pangenotypic regimens [26, 28].

### Address Barriers and Social Determinants

Several models effectively addressed social determinants of health that affect treatment uptake and adherence among PWUD. Common among these models was the use of multidisciplinary teams to engage and support patients during hospitalization and postdischarge. The 3 US models each integrated social work and/or case management to assess patient needs and treatment readiness during admission, which facilitated continuous engagement and addressed logistical challenges such as transportation and patient-provider communication [23, 24, 27]. Babiarz et al provided prepaid phones and offered temporary housing solutions, addressing the significant issue of housing instability [24]. Midgard et al recognized housing instability as negatively affecting treatment completion and identified in a subanalysis that early treatment initiation had a stronger impact on patients with unstable housing (interaction *P* = .004) [22].

### Outpatient Support as a Component of HCV Treatment Models

Postdischarge outreach that was versatile and supportive contributed to continued engagement in inpatient-initiated HCV treatment. Along with prepaid phones to aid in telehealth follow-up, Babiarz et al delivered medication refills to patients to minimize transportation barriers and to combat socioeconomic factors that negatively affect adherence [24]. Midgard et al and Le et al shared a more structured approach with dedicated follow-ups to local HCV clinics [22, 27]. McCrary et al utilized the simplified treatment approach to follow-up and monitoring, even obtaining SVR12 results opportunistically through interactions with patients at unrelated health care visits, such as emergency department encounters [23, 29, 30]. More structured outpatient follow-up models and minimal monitoring strategies both achieved high cure rates by creating opportune touchpoints across health care systems.

## LIMITATIONS

Since this was a narrative review, we did not evaluate the rigor of the study methodologies, which may introduce selection bias. Instead, we identified patterns in hospital-based HCV treatment models, focusing on how each model addressed established barriers for PWUD and influenced treatment initiation, completion, and SVR12 rates. Direct comparisons between treatment models are limited by variations in study design, patient populations, health care system structures, and access to DAAs. Differences in inclusion criteria and outcome definitions across studies further complicate direct comparisons. For example, Le et al reported high SVR12 rates but selectively excluded patients perceived as being less likely to complete therapy, whereas other studies included all eligible patients, even those who never initiated treatment [22–24, 27]. These discrepancies highlight that our aim was not to determine which model was superior but rather to assess the diverse ways that treatment models were structured to overcome barriers to care. The generalizability of findings may be limited by differences in health care policies, insurance coverage, and treatment access across regions and countries. The sample sizes of inpatient treatment initiations in most studies were small and highlight the need for more robust, larger, and multisite trials.

## BEST PRACTICES FOR INPATIENT HCV TREATMENT MODELS

- Optimize HCV identification systems to perform early screening and rapid confirmatory testing without HCV genotyping.
- Initiate DAA therapy during inpatient admission to reduce delays in the care cascade and increase treatment completion.
- Advocate for widespread availability of affordable pangenotypic DAA therapy. Multidisciplinary approaches, including the involvement of inpatient and outpatient pharmacists and social workers, are essential for effectively navigating the insurance and financial challenges of DAA procurement outside a single-payer system.
- Collaborate with multidisciplinary teams to address specific social and structural determinants of health among PWUD to reduce barriers to treatment adherence after discharge.
- Adopt low-barrier and simple approaches, such as telehealth services or minimal monitoring, to coordinate follow-up and facilitate engagement in care.

## FUTURE DIRECTIONS

HCV diagnosis and treatment during hospitalization has the potential to aid in global HCV elimination efforts. Future inpatient HCV treatment models that integrate simplified diagnostics, advocate for low-barrier DAA dispensation, and institute best practices from the multidisciplinary hospital-based models outlined here could have the greatest impact. Future opportunities include widespread adoption of point-of-care testing in the inpatient setting to accelerate identification of HCV so that treatment can be arranged even during short hospital admissions. Fingerstick point-of-care assays also offer a rapid and less stigmatizing approach to diagnosis for individuals who may have difficulties with venous access, such as PWUD [31]. Colocalizing social determinants of health and HCV treatment assessments during hospitalization could streamline the system of referrals and reduce drop-offs in the HCV care cascade. Eliminating restrictions to DAA distribution and reimbursement for inpatient pharmacies is key for hospital-based HCV treatment models to succeed. Models established in health care systems with universal coverage reported fewer, if any, barriers around DAA medication procurement for patients [22, 25, 27]. Last, outpatient-based models that have implemented peers (persons with lived experience) have demonstrated improved initiation of HCV treatment and viral clearance among PWUD [32, 33]. None of the hospital-initiated models in this narrative review utilized peers, which is a needed area of future study given the potential of integrating peer support into the inpatient treatment setting to smooth transitions of care and promote engagement in HCV treatment [34].

## CONCLUSION

Models for HCV treatment have significantly improved over the last decade by focusing on increasing diagnosis and retention in care, but innovative strategies are still necessary for global eradication. PWUD are a uniquely vulnerable population with barriers to linkage to care in traditional outpatient treatment models. Our review of hospital-initiated HCV treatment for PWUD offers an innovative and promising outline for future treatment model development that colocalizes timely diagnosis with linkage to social supports while placing HCV treatment in the hands of critically underserved populations.
